# Severe morbidity and mortality in untreated HIV-infected children in a paediatric care programme in Abidjan, Côte d'Ivoire, 2004-2009

**DOI:** 10.1186/1471-2334-11-182

**Published:** 2011-06-23

**Authors:** Sophie Desmonde, Patrick Coffie, Edmond Aka, Clarisse Amani-Bosse, Eugène Messou, François Dabis, Ahmadou Alioum, Andrea Ciaranello, Valériane Leroy

**Affiliations:** 1 Inserm, U897 & Institut de Santé Publique, Epidémiologie et Développement (ISPED), Université Victor Segalen Bordeaux 2, Bordeaux, France; 2 Programme PAC-CI, CHU de Treichville, Abidjan, Côte d'Ivoire; 3 CePReF, ACONDA, Abidjan Côte d'Ivoire; 4 MTCT-Plus Initiative, ACONDA, Abidjan, Côte d'Ivoire; 5 Massachusetts General Hospital, Boston, Massachusetts, USA

## Abstract

**Background:**

Clinical evolution of HIV-infected children who have not yet initiated antiretroviral treatment (ART) is poorly understood in Africa. We describe severe morbidity and mortality of untreated HIV-infected children.

**Methods:**

All HIV-infected children enrolled from 2004-2009 in a prospective HIV programme in two health facilities in Abidjan, Côte d'Ivoire, were eligible from their time of inclusion. Risks of severe morbidity (the first clinical event leading to death or hospitalisation) and mortality were documented retrospectively and estimated using cumulative incidence functions. Associations with baseline characteristics were assessed by competing risk regression models between outcomes and antiretroviral initiation.

**Results:**

405 children were included at a median age of 4.5 years; at baseline, 66.9% were receiving cotrimoxazole prophylaxis, and 27.7% met the 2006 WHO criteria for immunodeficiency by age. The risk of developing a severe morbid event was 14% (95%CI: 10.7 - 17.8) at 18 months; this risk was lower in children previously exposed to any prevention of mother-to-child-transmission (PMTCT) intervention (adjusted subdistribution hazard ratio [sHR]: 0.16, 95% CI: 0.04 - 0.71) versus those without known exposure. Cumulative mortality reached 5.5% (95%CI: 3.5 - 8.1) at 18 months. Mortality was associated with immunodeficiency (sHR: 6.02, 95% CI: 1.28-28.42).

**Conclusions:**

Having benefited from early access to care minimizes the severe morbidity risk for children who acquire HIV. Despite the receipt of cotrimoxazole prophylaxis, the risk of severe morbidity and mortality remains high in untreated HIV-infected children. Such evidence adds arguments to promote earlier access to ART in HIV-infected children in Africa and improve care interventions in a context where treatment is still not available to all.

## Background

In 2008, UNAIDS reported 14% of new human immunodeficiency virus (HIV) infections occurring in children below 15 years of age throughout the world [1]. By the end of 2008, it was estimated that 91% of HIV-infected children lived in sub-Saharan Africa [1]. Before ART was introduced, infant mortality of HIV-infected children on the African continent was high and premature, reaching 35% among children less than 12 months of age and 52% among children aged less than 24 months [2]. While the rate of new paediatric infections has slowly declined, only 38% of infected children have access to antiretroviral therapy (ART), with a huge heterogeneity between world regions [3]. In Côte d'Ivoire, the paediatric pandemic is sustained by a high prevalence of HIV among pregnant women (6.4%) [1], who, despite increasing access to mother-to-child transmission prevention (PMTCT) programmes, transmit the disease to their children [4].

In addition, providing a continuum of care between postnatal HIV diagnosis and paediatric care remains challenging, especially in Côte d'Ivoire [3-6], and the uptake of early infant diagnosis remains fairly poor. Thus, children are diagnosed belatedly, and when the HIV diagnosis is eventually made, the antiretroviral needs are not sufficiently met, estimated to reach only 15% in Côte d'Ivoire [1], leading to a severe morbidity and premature mortality before any access to ART [7-10].

In Africa, data on the disease progression of HIV-infected children before access to ART are rare, fragmented and most often obtained from selected children who have survived early opportunistic pathologies and who are therefore selected for these reasons [7-14]. In addition, the scarce existing data were collected during time periods when ART access was limited or nonexistent, when practices of care may have differed from the current standards of care in Côte d'Ivoire[11].

Detailed and accurate data on clinical evolution of HIV-infected children not on ART are essential in order to evaluate the effect of HIV care and treatment now being provided and to measure its effectiveness in the field as ART access is being scaled up [15]. In this paper, we describe the clinical evolution of HIV-infected children who have not yet initiated ART, assessed by severe morbidity and mortality and its determinants before access to ART, in a paediatric HIV-care programme in Abidjan, Côte d'Ivoire.

## Methods

### Study design and participants

In 2001, the Aconda Programme, a non-governmental association comprised mainly of health professionals, was implemented in Côte d'Ivoire. The main objective of this programme is to provide HIV and antiretroviral care in HIV-infected patients (adults and children) [3,16]. In 2004, Aconda, in partnership with the Institute of Public Health, Epidemiology and Development (ISPED, Bordeaux, France), launched a five-year programme of access to HIV care and treatment. ART was delivered according the 2006 WHO guidelines. The paediatric Aconda programme relies mostly on the two following HIV care centres: the paediatric CePReF site (*Centre de Prise en charge, de Recherche et de Formation*) which follows-up one of the largest active paediatric ART cohorts in Abidjan [3] and the MTCT-*Plus *programme, entirely dedicated to paediatric care following the enrolment of HIV-infected pregnant and post-partum women. Children included in the latter are, as a consequence, diagnosed earlier than in other sites [17]. Our work was a retrospective study conducted within the prospective assembly of a cohort of HIV-infected children who had not yet initiated ART and who were being followed-up in one of the two model paediatric health facilities in the Aconda care programme in Abidjan. All children under 15 years of age with a medical record who had not yet initiated any form of ART other than PMTCT interventions and who were enrolled in the programme between 1^st ^January 2004 and 31^st ^December 2009 after a confirmed HIV diagnosis by positive PCR at any age - or by positive serology if aged over 18 months - were included in this study.

### Data collection

The data collection took place retrospectively, from the CePReF and MTCT pre-existing databases. However, the CePReF database was not thorough and most of the data were extracted from the medical files. A standardised data collection instrument was issued for this purpose. The following variables were recorded into a database: date of inclusion, sex, date of birth, parental HIV status, parental vital status, date and method of diagnosis, HIV-diagnosis, PMTCT history, feeding history; date, height and weight; dates and causes of hospitalisation; dates and reason for treatment for in-patient daycare; dates and AIDS-defining morbid events; dates and receipts for cotrimoxazole, date and motif for cancellation of the prophylaxis; date of ART initiation; dates and results of haematology and CD4 cell count and percentage; and current status (transferred, LTFU, on ART, deceased or still known to be alive and not treated by ART).

### Event classification during follow-up

Data regarding all clinical examinations, in-patient daycare, and hospitalisations were extracted from the patients' medical records. The morbid events were defined and classified in the following ways: cases of respiratory tract infections, malnutrition, diarrhoea, tuberculosis, chronic HIV-associated lung disease, anaemia, encephalopathy and other AIDS-defining events were defined according to the WHO case definitions of HIV surveillance [18]. Other frequent paediatric infectious diseases such as varicella-zoster infections, measles, hepatitis B and malaria were defined according to the Red Book of Paediatrics [19]. Clinical events belonging to stages 1&2 of the WHO classification were not considered, neither were traumatic injuries. We defined any morbid event as severe if it led to one of the following: hospitalisation, in-patient daycare for more than 48 hours, or death.

### Statistical analysis

Baseline categorical data are presented as frequencies (%), and continuous data as median (interquartile range, (IQR)). Baseline differences between age groups and immunodeficiency status, according to the 2006 WHO recommendations (<15% if aged < 5 years; <200/mm^3 ^if aged ≥ 5 years) [19], were compared with Chi-2 tests, Fisher's exact tests, t-tests or Wilcoxon's tests where appropriate. We used the 2006 guideline definitions to be consistent with the study period, 2004-2009. The follow-up period was defined as the time between enrolment in the Aconda programme (time origin) and the date of ART initiation or the closeout date (date of death or date of last known to be alive). Two outcomes were assessed during this follow-up period: 1) the first severe morbid event and 2) mortality. The distributions of time to these outcomes were estimated using competing risk survival analysis. ART initiation is a competing cause of failure since children under ART have a better chance of survival [20-22]. To avoid making unrealistic assumptions about the independence of the outcomes, we chose to use a cumulative incidence function (CIF) to estimate the probabilities for each [23]. We ran a multivariate competing risks analysis, performed by the Fine and Gray proportional subdistribution hazard regression model. We used the package *cmprsk *of R statistical software version 2.11.1 (*The R foundation for Statistical computing, Vienna, Austria*) following guidelines presented by Scrucca *et al *[24,25]. If patients were lost to follow-up, data were right-censored at the date on which patients were last known to be alive (a patient was considered lost to follow-up after a 6 month period since the last clinical encounter).

Two multivariate models were estimated. In addition to age, immunodeficiency status, and gender, the other variables were inserted in the first "full" multivariate model if their p-value was less than 0.25. A second, "reduced" multivariate model was obtained from these same variables using descending stepwise procedures. The adjusted subdistribution hazard ratios were reported with their 95% confidence intervals (95%CI). Variables for which fewer than 70% of the patients provided data were not included. A p-value less than 0.05 was considered statistically significant on multivariate analysis in both "full" and "reduced" models.

### Ethical aspects

The national ethics committee of Côte d'Ivoire approved the Aconda programme data management system

## Results

**Figure 1:**
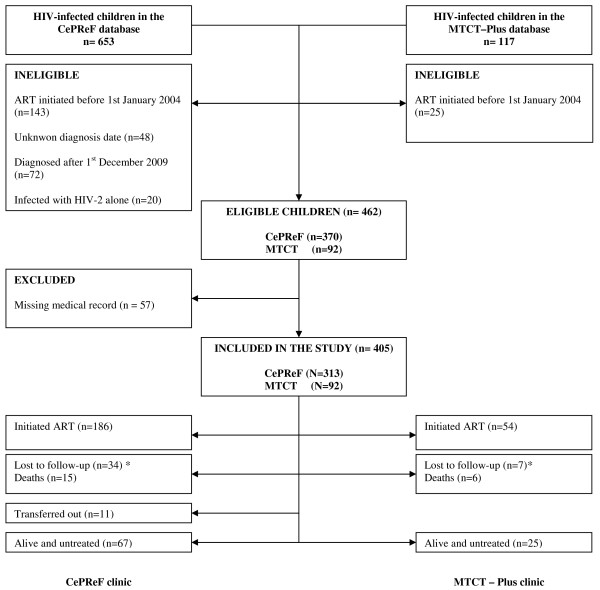
**Cohort profile of children included in the paediatric Aconda programme**. Children followed up at the CePReF and in the MTCT-Plus programme, Abidjan, Côte d'Ivoire (January 2004 - December 2009) *Missing data = date of HIV diagnosis unknown or missing medical record; †Lost to Follow-up: last clinical contact > 6 months.

Overall, 462 children fulfilled the inclusion criteria (Figure 1). Of these children, 57 (12.3%) were excluded because no medical record could be located, leaving 405 children included in the analysis.

### Characteristics of patients at baseline

The baseline characteristics of the 405 included children are presented in Table 1. Children were diagnosed at a median age of 4.5 years (IQR: 1.9; 7.5), and 47% were females. The median age of enrolment was lower amongst the children followed up in the MTCT programme (1.7 years (IQR: 0.1;4.9) vs 5.4 years (IQR:2.7;8.1), p <0.0001). Cotrimoxazole prophylaxis had been prescribed to 67% of the children before enrolment in the programme. The median weight for age z-score (WAZ) was -2.09 (IQR: -3.45;-0.95) among the children for whom anthropometric data were available. Approximately 50% of these children were suffering from failure to thrive (FTT), defined as a WAZ < -2. Immunological data (CD4 percent or count) were available for 308 children (76.1%). Of these children, 27.7% met the 2006 WHO criteria for immunodeficiency by age. The proportion of immunosuppressed children was highest amongst the 10-15 year olds (55%, p <0.0001).

**Table 1:** Characteristics by age in untreated HIV-infected children.

	Overall		Age < 1 year		Age 1 - 2 years		Age 2 - 5 years		Age 5 - 10 years		Age 10 - 15 years	
**BASELINE**	**N = 405**		**N = 64**		**N = 42**		**N = 108**		**N = 142**		**N = 49**	

**Age, years, median (IQR)**	4.5	(1.9; 7.5)	0.3	(0.01; 0.6)	1.7	(1.6; 1.8)	3.4	(2.6; 4.1)	7	(5.9; 8.5)	12.1	(11.2; 13.1)
**Female, n (%)**	189	(46.7)	30	(46.9)	19	(45.2)	47	(43.5)	75	(52.8)	18	(36.7)
**Weight available, n (%)**	383	(94.6)	59	(92.2)	37	(88.1)	100	(92.6)	138	(97.2)	49	(100.0)
**Height available, n (%)**	369	(91.1)	59	(92.2)	36	(85.7)	95	-88	131	(92.3)	48	(97.9)
**Weight for age z-score, median (IQR)**	-2.09	(-3.45; -0.95)	- 1.94	(-3.01; -0.91)	-2.67	(-4.10; -1.60)	-1.93	(-3.36; -0.74)	-1.84	(-3.22; -0.75)	-2.58	(-4.05; -1.62)
**Height for age z-score, median (IQR)**	-1,59	(-2;69; -0.76)	-		-1.34	(-1.47; -0.93)	-1.79	(-3.01; -0.74)	-1.32	(-2.44; -0.72)	-1.88	(-2.79; -1.06)
**Weight for height z-score, median (IQR)**	-1,11	(-2.63; -0.21)	-		-1.33	(-1.32; -0.38)	-0.86	(-2.37; 0.06)	-1.38	(-3.57; -0.43)	-1.67	(-1.85; -1.49)
**Failure to thrive, n (%)**	198	(51.7)	29	(49.2)	26	(70.3)	48	-48	63	(45.7)	32	(65.3)
**CD4 count measurement available, n (%)**	308	(76.1)	36	(56.3)	36	(85.7)	92	(85.2)	106	(74.6)	38	(94)
**CD4 cell %, median (IQR)**	19	(14; 25.5)	18.3	(14.1; 26)	17.9	(11.7; 23.3)	20.7	(14; 26.2)	-		-	
**CD4 count/μL, median (IQR)**	403	(151; 665)	-		-		-		507.5	(234; 723)	213.5	(55; 413)
Immunodepressed* children*, n (%)	112	(27.7)	10	(15.6)	10	(15.6)	26	(24.1)	39	(27.5)	27	(55.1)
**CDC stage C, n (%)**	50	(12.3)	7	(10.9)	6	(14.3)	12	(11.1)	14	(9.9)	11	(2.1)
**Baseline cotrimoxazole prophylaxis n (%)**	271	(66.9)	23	(35.9)	33	(78.6)	74	(68.5)	102	(71.8)	39	(67.4)
**Born to known HIV-infected mothers**	273	(67.4)	64	(100)	33	(78.6)	86	(76.6)	77	(54.2)	13	(26.5)
**Diagnosis by PCR before 18 months**	71	(19.2)	53	(82.8)	6	(14.3)	5	(4.6)	6	(4.2)	1	(2.1)
**Mother deceased at inclusion, n (%)**	97	(30.9)	1	(1.6)	1	(2.4)	15	(13.9)	52	(36.6)	28	(57.1)
**Breastfeeding history, n (%)**	191	(47.2)	40	(62.5)	19	(45.2)	43	(39.9)	67	(47.2)	22	(44.9)
**History of PMTCT intervention**	65	(18.1)	50	(78.1)	10	(23.8)	5	(4.6)	-		-	

Children followed-up by the CePReF and the MTCT-Plus programme, Abidjan, Côte d'Ivoire (January-2004-December 2009). n = 405. * Immunodeficiency according to the 2006 criteria;

### Patient follow-up

Among the 405 children included, the overall median pre-ART follow-up time was 12 months (IQR: 1.3;30.6). Over the study period, 240 (59.1%) initiated ART, 21 (5.2%) died, 41 (10%) were lost to follow-up, 11 (2.7%) transferred to other healthcare facilities and 92 (23%) were still known to be alive and untreated at the endpoint date (Figure 1).

### Morbidity

**Figure 2:**
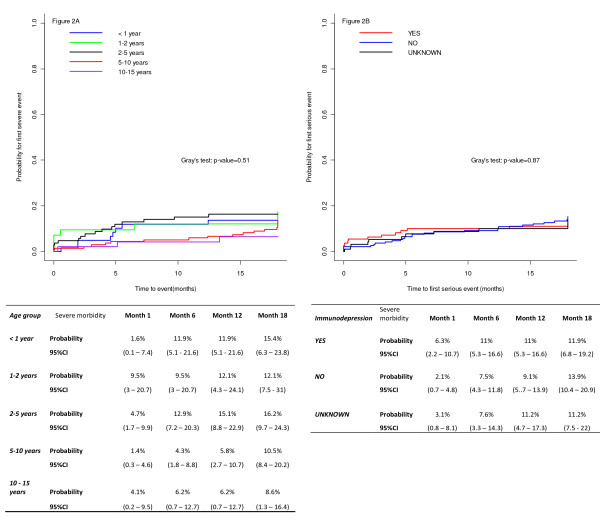
**Cumulative incidence functions for first severe morbidity by age (2A) and immunodeficiency status (2B)**. Children followed up at the CePReF and in the MTCT-Plus programme, Abidjan, Côte d'Ivoire (January 2004 - December 2009). n = 405.

Forty-two percent (170 children) presented with at least one HIV-related condition during follow-up. Fifty-six children (14%) presented with a first severe morbid event during the first 18 months of follow-up, most frequently malaria (37.5%) and WHO stage 3 events (28.3%). Of these severe morbid events, 16 (28.6%) led to death (Table 2). The observed incidence density rate of occurrence of the first severe event was 17.8 per 100 child-years of follow-up. From the date of enrolment in the programme, the overall median time of infection until occurrence of the first severe event was 9.1 months (IQR: 1.2;26.3). The probability of developing a first severe morbid event at 18 months was high, with an overall cumulative incidence of 11% (95%CI: 10.9;11.1). During these first 18 months of follow-up (Figure 2), the probability did not differ significantly by age at enrolment nor by immune status (p = 0.51 and p = 0.87 respectively). Univariate analysis show that children in the MTCT programme were less at risk of a first severe event occurring than those in the CePReF programme (sHR: 0.43, 95%CI:0.19;1), (Table 3). However, this association did not remain in the full multivariate model (Table 3), due to correlation between the age and programme variables (children in the MTCT programme were younger). Associations between baseline characteristics and the occurrence of the first severe event were not seen in the full model, but the reduced model suggested a lower risk in children ≥5 years of age at enrolment compared to those <5 years of age (5-10 years, sHR: 0.30, 95%CI: 0.10;0.97 and 10-15 years, SHR: 0.15, 95%CI:0.03;0.66). This association was confounded by PMTCT interventions and immunodeficiency status, however. The reduced model also showed a lower risk in children previously exposed to any kind of PMTCT intervention (sHR: 0.16, 95%CI: 0.04;0.71, versus those not exposed).

**Table 2:** Distribution of the first severe morbid event in untreated HIV-infected children

Diagnosis	Number of first events, n*(%)*		Lethal events, n*(%)*	
***WHO Stage 2***				
Hepatosplenomegaly	1	*(1,7)*	-	
				
***WHO Stage 3***				
Failure to thrive (FTT)	2	*(3,6)*	1	*(50)*
Unexplained persistent diarrhoea	4	*(7,1)*	-	
Unexplained persistent fever	1	*(1,7)*	-	
Tuberculosis	1	*(1,7)*	1	*(100)*
Chronic lung disease	4	*(7,1)*	-	
Severe bacterial infections	4	*(7,1)*	2	*(50)*
				
***WHO Stage 4***				
Pneumonia	1	*(1,7)*	-	
				
***Other diagnosis of interest***				
Malaria	21	*(37,5)*	-	
Meningitis	2	*(3,6)*	2	*(100)*
				
***Unknown***	15	*(26,7)*	10	*(67)*
				

***Total***	56	*(100)*	16	(28,6)

Children followed-up over 18 months by the CePReF and the MTCT-Plus programme, Abidjan, Côte d'Ivoire (January-2004-December 2009). n = 405.

**Table 3:** Characteristics associated with the first severe morbidity during follow-up in untreated HIV-infected children.

					Univariate analysis			Ajusted analysis					
					
								Full model			Reduced model		
								
Variable		# patients	# events	*Observed rate (/100 CY)*	sHR†	95%CI‡		*sHR*	*95%CI*	*P*	sHR	95%CI	*P*
**Overall**		**405**	**58**	*17.80*									
													
**Age at baseline (years)**							***0.54***			***0.39***			***0.07***
	< 1	64	7	12.78	1	-		1	-		1	-	
	[1 - 2]	42	7	20.36	1.29	*(0.47-3.58)*		0.45	*(0.12 - 1.64)*		0.52	*(0.13 - 2.01)*	
	[2 - 5]	108	17	20.41	1.24	*(0.53 - 2.91)*		0.43	*(0.14 - 1.31)*		0.42	*(0.14 - 1.32)*	
	[5 - 10]	142	21	16.65	0.94	*(0.41 - 2.15)*		0.38	*(0.13 - 1.14)*		0.30	*(0.10 - 0.97)*	
	[10 - 15]	49	4	14.58	0.45	*(0.12 - 1.70)*		0.23	*(0.05 - 1.02)*		0.15	*(0.03 - 0.66)*	
**Immunodeficiency**							***0.87***			***0.91***			***0.86***
	No	112	29	13.61	1	-		1	-		1	-	
	Yes	106	15	39.24	0.84	*(0.43-1.63*		0.88	*(0.47 - 1.65)*		0.84	*(0.43 - 1.63)*	
	Unknown	98	14	18.75	0.91	*(0.46 - 1.77)*		1.02	*(0.54 - 1.93)*		1.03	*(0.51 - 2.07)*	
**Gender**							***0.78***			***0.95***			***0.77***
	Male	216	32	18.07	1	-		1	-		1	-	
	Female	189	26	17.46	0.93	*(0.54 - 1.60)*		0.91	*(0.54 - 1.58)*		0.91	*(0.51 - 1.61)*	
**History of a PMTCT intervention**							***0.11***			***0.19***			***0.05***
	No	267	41	21.72	1	-		1	-		1	-	
	Yes	65	5	7.41	0.43	*(0.15 - 1. 02)*		0.32	*(0.06 - 1. 63)*		0.16	*(0.04 - 0.72)*	
	Unknown	73	12	17.20	1.04	*(0.53 - 2.02)*		1.21	*(0.56 - 2.59)*		0.82	*(0.42 - 1.59)*	
**Mother known to be deceased**							***0.13***			***0.26***			
	No	308	48	18.19	1	-		1	-				
	Yes	97	10	16.10	1.78	*(0.84 - 3.14)*		1.51	*(0.73 - 3.09)*				
**Cotrimoxazole prophylaxis at baseline**							***0.35***						
	No	134	17	15.60	1	-							
	Yes	271	41	18.89	1.33	*(0.73 - 2.43)*							
**Follow-up Centre**							***0.05***			***0.14***			
	Cepref	131	50	21.92	1	-		1	-				
	MTCT	92	8	8.17	0.43	*(0.18 - 1)*		0.45	*(0.16 - 1.29)*				

Children followed-up over 18 months by the CePReF and the MTCT-Plus programme, Abidjan, Côte d'Ivoire (January-2004-December 2009). n = 405. Fine and Gray Model CY: Child Years; †sHR: subdistribution hazard ratio; ‡95% confidence interval

### Mortality

By the end of the study, 21 deaths were recorded, 90.5% of which occurred at home; the median time to death was 12 months (IQR:1.5;30.8) and the incidence density rate was estimated as 3.26 per 100 child-years of follow-up (95%CI: 3.12;3.39). Fifty-two percent of the deaths occurred in children aged less than 2 years. The mortality rates for children aged 0-1 and 1-2 years were 9.70 and 3.41 per 100 child-years of follow-up, respectively (Table 1).

**Figure 3:**
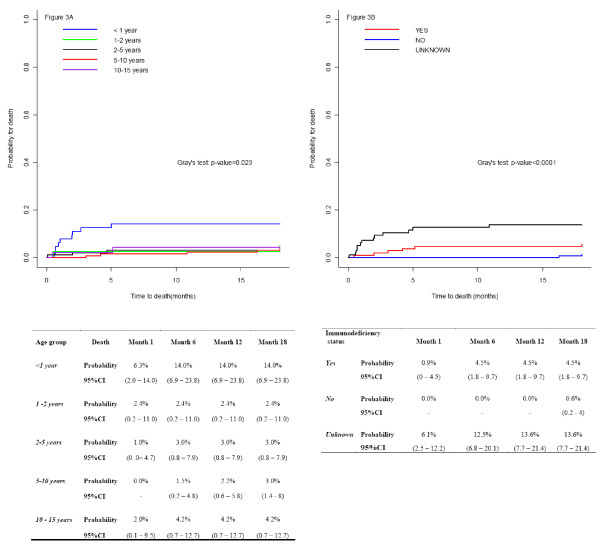
**Cumulative incidence functions for mortality by age (3A) and immunodeficiency status (3B)**. Children followed up at the CePReF and in the MTCT-Plus programme, Abidjan, Côte d'Ivoire (January 2004 - December 2009). n = 405

The overall cumulative mortality reached 5.5% (95%CI: 5.3;5.7) at 18 months; reaching 14% at 18 months of follow-up among children less than 12 months (p = 0.023) (Figure 3A). Immunosuppressed children also had a higher probability of death at 18 months compared to non-immunosuppressed children (4.5%, 95%CI: 1.8;9.7, vs 0.6%; 95%CI: 0.2;4.0). In addition, the absence of immunological data was informative; children with unknown immunologic status had the highest risk of death by 18 months: 13.6% (95%CI: 7.7;21.4; Figure 3B).

Adjusted for gender and immune status, age was no longer associated with death (p = 0.07), but immune-supression remained a significant predictor of death (sHR: 6.02, 95% CI: 1.28;28.42), (Table 4).

**Table 4:** Characteristics associated with mortality during follow-up in untreated HIV-infected children.

					Univariate analysis			Ajusted analysis					
					
								Full model			Reduced model		
								
Variable		# patients	# events	*Observed rate (/100 CY)*	sHR†	95%CI‡	*P*	*sHR*	*95%CI*	*P*	sHR	95%CI	*P*
**Overall**		**405**	**21**	3.26									
													
**Age at baseline (years)**							***0.034***			***0.840***			***0.097***
	< 1	64	9	9.70	1	-		1	-		1	-	
	[1 - 2]	42	2	3.40	0.32	*(0.07 - 1.48)*		0.76	*(0.08 - 6.38)*		0.6	*(0.11 - 3.23)*	
	[2 - 5]	108	3	1.68	0.2	*(0.05 - 0.74)*		0.44	*(0.07 - 2.70)*		0.34	*(0.08 - 1.39)*	
	[5 - 10]	142	5	1.82	0.24	*(0.08 - 0.71)*		0.39	*(0.06 - 2.31)*		0.29	*(0.10 - 0.80)*	
	[10 - 15]	49	2	4.91	0.28	*(0.06 - 1.29)*		0.44	*(0.05 - 4.26)*		0.28	*(0.06 - 1.19)*	
**Immunodeficiency**							***0.001***			***0.003***			***0.0025***
	No	112	6	1.36	1	-		1	-		1	-	
	Yes	106	2	3.38	5.21	*(1.00 - 25.60)*		6.8	*(1.51 - 30.57)*		6.02	*(1.28 - 28.42)*	
	Unknown	98	13	8.90	13.82	*(3.16 - 60.50)*		12.93	*(2.95 - 56.68)*		12.6	*(2.94 - 53.97)*	
**Gender**							***0.920***			***0.770***			***0.670***
	Male	216	11	3.09	1	-		1	-		1	-	
	Female	189	10	3.45	1.05	*(0.47 - 2.45)*		1.15	*(0.44 - 2.98)*		1.22	*(0.49 - 3.04)*	
**History of a PMTCT intervention**							***0.019***			***0.590***			
	No	267	11	2.80	1	-		1	-				
	Yes	65	8	6.73	2.96	*(1.19 - 7.33)*		0.92	*(0.14 - 6.21)*				
	Unknown	73	2	1.48	0.63	*(0.14 - 2.83)*		0.47	*(0.11 - 2.05)*				
**Mother known to be deceased**							***0.140***			***0.230***			
	No	308	19	3.59	1	-		1	-				
	Yes	97	2	1.72	3.01	*(0.71 - 12.80)*		2.78	*(0.53 - 14.68)*				
**Cotrimoxazole prophylaxis at baseline**							***0.150***			***0.830***			
	No	134	11	4.93	1	-		1	-				
	Yes	271	10	2.36	0.53	*(0.23 - 1.25)*		1.13	*(0.37 - 3.43)*				
**Follow-up Centre**							***0.550***						
	Cepref	131	15	3.34	1	-							
	MTCT	92	6	3.05	1.33	*(0.52 - 3.45)*							

Children followed-up over 18 months by the CePReF and the MTCT-Plus programme, Abidjan, Côte d'Ivoire (January-2004-December 2009). n = 405. Fine and Gray Model. CY: Child Years; †sHR: subdistribution hazard ratio; ‡95% confidence interval

## Discussion

This cohort study from Abidjan, Côte d'Ivoire, documents patterns of severe morbidity and mortality in HIV-1 infected children before the initiation of ART, in a paediatric care programme in which ART access was being scaled-up. There are two primary findings. First, the risk of severe morbidity before ART initiation is high, reaching 11% at 18 months. The risk of first serious morbidity was minimised in older children but also by having received any intervention for PMTCT. Second, the risk of mortality before ART initiation is high (reaching 8% at 18 months), and this risk is greater in immunosuppressed children and children with unavailable immunological data. Importantly, the results from this cohort study may underestimate the true risks of morbidity and mortality, as they were derived from a selected population included in research programmes, where healthcare support might have been better than that offered outside of the Aconda context.

In addition, our results highlight the impact of age at enrolment. The overall incidences of severe morbidity after 18 months of follow-up do not vary by immune status but by age, which is consistent with a rapid CD4 evolution in the younger children. As the children followed up in the MTCT programme are significantly younger, there is a strong correlation between age and follow-up centre; we therefore chose, in the reduced model, to exclude the centre variable from the analyses. Adjusting for PMTCT, immunosupression and gender had an effect on the age variable in the final reduced model; children aged ≥5 were less at risk of presenting with a first morbid event. Exposure to PMTCT interventions was more frequent among the younger children and therefore the two variables were confounded. Based on previous studies reporting PMTCT interventions as having a protective effect on severe morbidity [26,27], we expected to observe a lower risk of severe morbidity among the younger children. However, our study shows higher risks among these children. We hypothesise that the outcomes of PMTCT interventions are not the only explanation for the observed results, but the available data does not allow further conclusions; PMTCT is a marker of early and different access to care for younger children than for older children. We show here the positive effect of early access to care on reducing the risk of occurrence of the first severe HIV-related morbid event.

These results highlight the importance of comprehensive HIV care in addition to ART. Despite prophylaxis by cotrimoxazole at baseline to prevent opportunistic infections [28], substantial morbidity attributable to other co-infectious disease (bacterial, viral and parasitic infections) was observed. Optimal prevention and care for comorbidities needs to be investigated before and after ART access to improve paediatric care.

Mortality was moderate in this cohort, reaching 8% at 18 months. Although mortality risk was significantly higher among infants < 12 months than among older infants in univariate analyses, we found no overall significant effect of age in multivariate analyses. There was, however, a 12-fold increase in mortality risk for children who had no CD4 measurement. Infants aged < 12 months have previously been reported to have higher risk for death and loss to follow-up than older children [6]; among infants <12 months old in our cohort, 50% had no available CD4% data at baseline. This correlation between young age and unavailable CD4% partially explains the observed increased risk of mortality in children without CD4% data. The reasons for this lack of CD4% availability in younger children are also critical. Children diagnosed as HIV-infected and then enrolled in the clinic often have to return on a different date for CD4 measurement if no staff members are available for phlebotomy at the initial visit. While most of these children do return, many others are lost to follow-up. Reasons for loss-to-follow-up remain a primary research question in paediatric care, but in light of these results, we can hypothesise that many deaths occurring at home during the interval before the scheduled return visit, and then are reported to the clinic at a later date when contacted by a care worker. This demonstrates the critical need for improved and expedited linkage to care between diagnosis, CD4% measurement, and ART initiation.

There are two important limitations inherent in our study design. First, the timing of paediatric HIV infection could not be known with complete accuracy. In Abidjan, like in most resource limited settings, social barriers and stigma associated with HIV/AIDS often lead to delays in accessing care and in receipt of HIV diagnosis. The youngest children in our study were most likely infected through MTCT. Although the exact time of infection (antepartum, intrapartum, or postnatal) is not documented, we could reasonably estimate the time of infection as being the date of birth. However, older children in this cohort may have been infected perinatally, or they may have been exposed to HIV by blood transfusion, sexual activity or hospital-acquired infections. These children are mostly followed up in the CePReF programme, having been diagnosed with HIV-1 following a severe opportunistic infection; their time of infection cannot be estimated with the available data. Furthermore, mortality is severe amongst infants in the absence of ART [4], which combined with the late diagnoses issues, induces a strong left truncation phenomenon [29]. To address this left-censoring of the data, we modelled infection from the date of enrolment in the healthcare programme, stratifying by age. Thus, the information observed over different age groups provides a detailed estimate of clinical disease progression in prevalent cohorts for older children, but could be considered as the clinical disease progression of perinatally infected children for those aged less than 5 years.

Second, morbidity and mortality may have been incompletely ascertained. Many African families withhold information concerning infection and diagnosis, as well as information relative to prior events and deaths during follow-up [30]. In addition, missing data arose as a result of the reality of healthcare services in the field in Africa and the lack of follow-up in clinical examinations. The data coming from the pre-existing MTCT datasets were precise, as they derive from previous research programmes, but most of the data from the CePReF, which represents 77% of the children, was collected retrospectively by the means of a standardised collection instrument. Record-keeping practices may vary over time and between providers. As a consequence, diagnoses could not be confirmed using standardised diagnosis procedures. We attempted to ensure the accuracy of clinical events by requiring documentation, such as clinical findings, laboratory examinations, and radiographic results. However, this may have led to under-documentation of the true rate of clinical events. This could explain, in part, the observed lower risk of severe morbidity in the older children. Moreover, the morbidity observed must be interpreted with caution. Not only are the diagnoses not confirmed, but there is no control group of non-HIV-infected children with whom to compare specific morbidity rates. Nonetheless, we show here a high rate of overall severe and avoidable morbidity despite prophylaxis in HIV-infected children who do not have access to ART.

In addition to morbidity, the infant mortality rate in this cohort may be underestimated. Many events leading to death remain undetermined. These unknown causes of death are frequent because most deaths occurred at home; these deaths were most likely reported to the clinic only in response to a follow-up call made by a care worker. We also suspect that a substantial proportion of the children lost to follow-up are in fact deceased. Moreover, many of these children were immunosuppressed according to the 2006 WHO criteria, which is a known predictor of death. Nevertheless, the overall loss to follow up rate was only 11% at 18 months; even taking into account the exclusion of 12.3% of the eligible cohort for missing medical records (perhaps also representing loss-to-follow-up), this rate of missingness is low in the African context compared to those of cohorts of children on ART [3,15,31].

Our study is the first to investigate pre-ART morbidity and mortality in a large paediatric cohort with a wide age distribution during the ART era, reflecting as well as possible current care practices in Côte d'Ivoire [8,32-34]. Several of our results are in keeping with previous findings, suggesting the validity of our results despite the limitations described above. First, the mortality documented in this cohort is in accord with the bi-modal pattern of the paediatric HIV infection, mainly characterised by early mortality. We report a mortality rate three times higher in children aged less than 12 months than in older children, but the numbers remain low compared to those of previous studies [34-37]. As have shown recent studies, CD4 cell counts are important indicators [38,39] of mortality in this analysis.

Our study first suggests low rates of death but high rates of severe morbidity among non-ART treated HIV infected children. Despite the efforts of implementing antiretroviral access and PMTCT interventions in Abidjan, our results demonstrate still a high rate of infected children who do not have access to ART. This justifies the revision of the WHO guidelines recommending ART to all infected children aged < 24 months regardless of CD4 count, but this is not always applied in the field. In many resource-limited settings, ART coverage remains low mainly for operational reasons, leading to high mortality and morbidity. Additional paediatric clinical research must be undertaken in order to measure the incidence of mortality, morbidity, and loss to programme in infected children before and after ART access and the use of prophylactic treatment in opportunistic infections. This would allow investigators to model the evolution of the paediatric infection and measure the cost effectiveness of interventions with a final goal to guiding care of children infected by HIV in Africa, as has been done successfully in the past for adults [40-42].

## Conclusions

We describe clinical progression in untreated, HIV-infected children in Abidjan, Côte d'Ivoire, in the context of paediatric-specific HIV care. Having benefited from early access to care minimizes the severe morbidity risk for those children who acquire HIV. Despite the receipt of cotrimoxazole prophylaxis, the risk of serious morbidity and mortality remains high in ART-untreated HIV-infected children. As such clinical events are likely preventable by early initiation of ART to improve immune status, these findings demonstrate the need to promote an earlier access to ART in HIV-infected children in Africa, in a context where early ART is officially recommended and is being scaled up, but is still not available to a majority of children.

## Competing interests

The authors declare that they have no competing interests.

## Authors' contributions

EA, CB and EM contributed the necessary data from their health facilities. SD collected the data. PC, EA, CB, EM and VL contributed to the study design. SD, AA and VL carried out the statistical analysis and interpreted the results. FD, AC and VL contributed to the coordination. SD and VL first drafted the manuscript. Critical revision of the manuscript for important intellectual content: SD, PC, FD, AA, AC and VL. All authors read and approved the final manuscript.

## Pre-publication history

The pre-publication history for this paper can be accessed here:

http://www.biomedcentral.com/1471-2334/11/182/prepub
